# Porphyrins‐Assisted Cocatalyst Engineering with Co—O—V Bond in BiVO_4_ Photoanode for Efficient Oxygen Evolution Reaction

**DOI:** 10.1002/advs.202206729

**Published:** 2023-01-16

**Authors:** Linxing Meng, Zunyan Lv, Weiwei Xu, Wei Tian, Liang Li

**Affiliations:** ^1^ School of Physical Science and Technology Jiangsu Key Laboratory of Thin Films Center for Energy Conversion Materials and Physics (CECMP) Soochow University Suzhou 215006 P. R. China

**Keywords:** BiVO_4_, cocatalysts, photoanode, water splitting

## Abstract

The application of photoelectrochemical (PEC) water splitting is limited by the sluggish surface oxygen evolution reaction (OER) kinetics. OER kinetics can be effectively improved through cocatalyst engineering. However, the tardy transfer process and serious recombination of carriers are the key factors restricting the cocatalyst development. Taking BiVO_4_ as an example, a Co‐modified heme film rich in large conjugated ring structures is introduced onto the photoanode surface using a solvothermal method. This film functions as an efficient cocatalyst. It considerably reduces the surface overpotential, promotes the transfer of photogenerated holes, and boosts the kinetics of OER by specifically affecting the formation of OOH*. Simultaneously, the formed Co—O—V bonds induce strong interaction at the photoanode/cocatalyst interfaces, reducing the recombination of photogenerated carriers. Consequently, the onset potential of the optimized photoanode decreases from 0.45 to 0.07 V and the photocurrent density at 1.23 V versus reversible hydrogen electrode boosts to 5.3 mA cm^−2^. This work demonstrates a facile strategy for designing cocatalysts to obtain rapid hole transfer capability and reduced carrier recombination for improved PEC performance.

## Graphical abstract

A simple solvothermal‐assisted process is utilized to introduce large conjugated ring structure‐rich cocatalyst films onto the surface of the BiVO_4_ photoanode. The covered films act as an efficient cocatalyst, effectively facilitating the transfer of holes, reducing the surface overpotential, and boosting the kinetics of surface oxygen evolution reaction by precisely affecting the forming processes of OOH*.

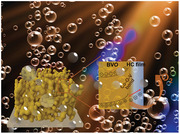

## 1 Introduction

Photoelectrochemical (PEC) water splitting is a promising technology to utilize solar light to produce clean hydrogen energy.^[^ ^1^, ^2^, ^3^ ^]^ Owing to the four‐electron transfer process, the sluggish oxygen evolution reaction (OER) kinetics at the photoanode primarily hinders PEC performance.^[^ ^4^, ^5^ ^]^ In general, elemental doping, loading of cocatalysts,^[^ ^6^ ^]^ passivation of surface defects,^[^ ^7^ ^]^ vacancy engineering,^[^ ^8^, ^9^ ^]^ and crystal face modification^[^ ^10^, ^11^ ^]^ can promote transfer of photogenerated carriers on the photoanode surface. Among these strategies, cocatalyst engineering is one of the most efficient means to enhance OER kinetics and improve the performance of the photoanode.^[^ ^12^ ^]^

Cocatalysts can address tardy OER kinetics by promoting the transfer of photogenerated holes, reducing the recombination of photogenerated carriers, and reducing the overpotential (*η*) of the surface OER.^[^ ^13^, ^14^, ^15^ ^]^ Nevertheless, the lack of hole transfer capability and a large amount of recombination prevent the improvement of photoanode performance during cocatalyst design.^[^ ^16^, ^17^ ^]^ NiOOH effectively promotes OER kinetics as a cocatalyst, however, its ability to capture photogenerated holes is insufficient. Furthermore, the hole transport resistance of FeOOH is lower than that of NiOOH. Therefore, Kim and Choi demonstrated that incorporating a FeOOH compound can accelerate hole transport from BiVO_4_ (BVO) to NiOOH and improve the performance of the BVO photoanode.^[^ ^18^ ^]^ Further, Park et al. solved this problem by introducing a layer of black phosphorene (BP) nanosheets that serves as an excellent hole extraction layer between BVO and the cocatalyst for solar water splitting.^[^ ^19^ ^]^ The existence of BP caused a photocurrent density (*J*) of 4.48 mA cm^−2^ at 1.23 V versus reversible hydrogen electrode (RHE). These works demonstrate that the water oxidation capability of cocatalysts is strongly restricted by their insufficient hole extraction from the photoanodes. Although the additional layers can facilitate the transfer of photogenerated holes, the introduction of layers complicates the fabrication of the device.^[^ ^20^, ^21^ ^]^ It even introduces additional recombination centers, resulting in severe carrier recombination.^[^ ^22^ ^]^ More specifically, cocatalysts are usually loaded using hydrothermal or spin coating methods.^[^ ^23^, ^24^ ^]^ This leads to poor interaction at the photoanode‐cocatalyst interfaces with many recombination sites and high electrical resistance, blocking the surface catalysis of photogenerated holes for participating in water oxidation reactions. Therefore, designing efficient cocatalysts with fast hole transfer capability and less carrier recombination using simple methods for enhancing PEC performance is important and challenging.

**Figure 1:**
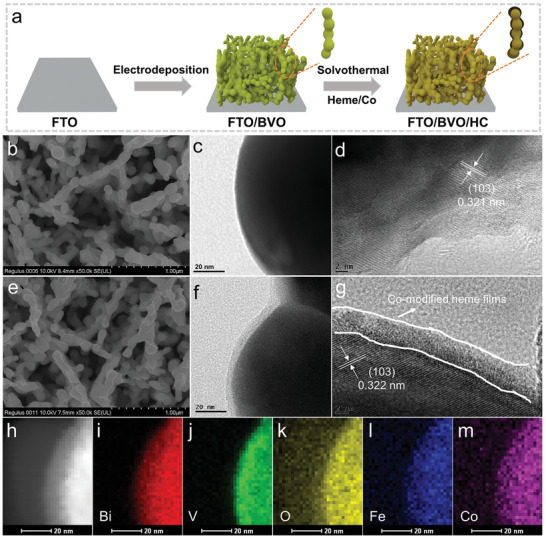
a) Schematic for the synthetic process of the photoanode. b,d) Top‐view SEM images of BVO and BVO/HC. c,f) TEM and d,g) HRTEM images of BVO and BVO/HC, respectively. h–m) Elemental mapping of BVO/HC.

Porphyrins are macromolecular heterocyclic compounds and have large conjugated ring structures with capability of quick photogenerated carrier transfer.^[^ ^25^, ^26^, ^27^ ^]^ As a classic Fe‐based porphyrin compound, heme (He) exhibits excellent hole extraction and transfer properties, making it a potential raw material for designing cocatalysts.^[^ ^28^, ^29^ ^]^ Here, we selected BVO as the photoanode material and loaded Co‐modified He (named HC) onto the surface of BVO using a simple solvothermal method. Co^2+^ was used to replace Fe^2+^ to build Co—O—V bonds at the photoanode/cocatalyst interface, affording the HC film‐coated BVO photoanode (named BVO/HC). The experimental process is shown in **Figure** **1**a. Experiments and theoretical calculations reveal that the HC film acts as an efficient cocatalyst, which considerably reduces surface *η*, promotes the transfer of photogenerated holes, and boosts OER kinetics by precisely affecting the formation of OOH*. Moreover, introducing the Co—O—V bonds enhance the photoanode‐cocatalyst combination and effectively reduces the recombination of carriers. Finally, the BVO/HC photoanode presents a considerably increased *J* of 5.3 mA cm^−2^ at 1.23 versus RHE and onset potential (*V*_on_, the potential at which 0.05 mA cm^−2^ was first measured) of 0.07 versus RHE in 0.5 M Na_2_SO_4_ aqueous, which is 4.4 times higher than that of BVO. The performance is better than other up‐to‐date reported BVO ‐based photoanodes (Table S1, Supporting Information).

## 2 Results and Discussion

**Figure 2:**
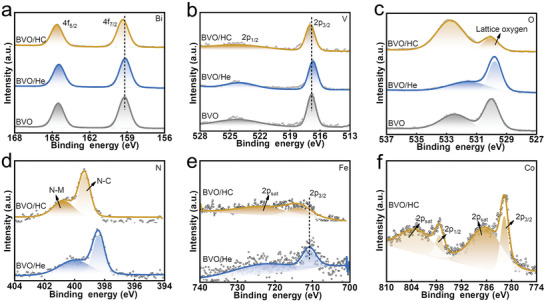
a) Bi 4f, b) V 2p, and c) O 1s XPS of BVO, BVO/He, and BVO/HC, respectively. d) N 1s, e) Fe 2p XPS for BVO/He and BVO/HC. f) Co 2p XPS of BVO/HC.

Through a facile solvothermal method, the HC films were introduced onto the surface of BVO. Scanning electron microscopy (SEM) and transmission electron microscopy (TEM) images have been applied to characterize the morphology of photoanodes. BVO and BVO/HC display similar morphology with worm‐like structure (Figure 1b–e). The energy‐dispersive X‐ray (EDX) element mapping under SEM reveals the elements of C, N, Fe, and Co distributed evenly in BVO/HC (Figure S1, Supporting Information), indicating that the HC film was successfully coated on the surface of BVO. TEM images further reveal that a thin film of ≈5 nm appeared on the surface of BVO/HC (Figure 1f) compared with BVO (Figure 1c), indicating that HC film was coated on the surface of BVO. X‐ray diffraction (XRD) analysis was employed to further investigate the crystal structures of the as‐prepared photoanode. BVO and BVO/HC exhibit similar diffraction peaks (Figure S2, Supporting Information) for BiVO_4_ (JCPDS: 83–1699),^[^ ^30^ ^]^ owing to the amorphous properties of HC films. Further, the high‐resolution TEM (HRTEM) image in Figure 1d reveals the crystal lattice spacing of 0.321 nm, corresponding to the (103) plane of BiVO_4_. BVO/HC shows the same (103) plane with a lattice spacing of 0.322 nm (**Figure** **2**g), demonstrating the loading of HC film has no effect on BVO. Additionally, an amorphous overlayer is observed on the BVO/HC photoanode (Figure 2g and Figure S3, Supporting Information), revealing that the HC film is supported on the surface of BVO in an amorphous form. Raman spectra in Figure S4 (Supporting Information) were measured to observe the molecular structure changing of BVO and BVO/HC. Raman spectra of BVO have distinctive vibrational modes ≈127, 212, 326, and 367 cm^−1^, which reflects VO_4_ tetrahedrons in the monoclinic BiVO_4_ system. While the peak position of BVO/HC changed a little compared with BVO, indicating that the presence of HC film affects V and the Co—O—V bond formed, which will be discussed in the following part. Additionally, the sharp Raman peaks at 1368, 1568, and 1633 cm^−1^ are observed for BVO/HC, corresponding to the C=C bending and C—C bending vibration peaks, respectively.^[^ ^31^ ^]^ The EDX element mapping images are shown in Figure 1h–m to figure out the element distribution of elements. The Bi, V, O, Fe, and Co elements exhibit a uniform distribution, reflecting that HC films are well covered on the surface of BVO. These data demonstrate that a conformal‐covered amorphous film is obtained on the surface of BVO using a simple solvothermal method.

X‐ray photoelectron spectroscopy (XPS) was used to detect the chemical composition and valence states of elements. To explore the state of introduced Co in the samples, we prepared Co‐free samples (named BVO/He) for comparison. As shown in Figure S5, Supporting Information), the XPS survey spectra prove the existence of Bi, V, and O elements in BVO, BVO/He, and BVO/HC. Nevertheless, after coating the surface with an HC film, the N, Co, and Fe elements appeared in BVO/HC, indicating the existence of Co‐modified He on the surface of BVO. The peak positions of BVO located at 164.52 and 159.23 eV are attributed to 4f_5/2_ and 4f_7/2_ of Bi^3+^, respectively (Figure 2a). The peaks of BVO at 524.18 and 516.86 eV in Figure 2b can be ascribed to 2p_1/2_ and 2p_3/2_ of V^5+^, respectively. Figure 2c shows the two peaks of O at 532.53 and 529.98 eV, which are related to the surface‐absorbed H_2_O and lattice oxygen, respectively. The peaks of Bi 4f were unchanged when BVO was coated with a He film, while the peaks of V 2p and O 1s in BVO/He shifted toward lower binding energy compared with those of BVO. We propose this is because the coated He film combined with BVO and formed Fe—O—V bonds. This process introduces defects and causes charge recombination around V and O, increasing electron cloud density. Similarly, compared with BVO/He, the peaks of V and O shifted toward higher binding energy in BVO/HC, which confirmed that the modification of Co influences the He film. Since the atomic radius of Co is smaller than that of Fe, the bond energy obtained by combining with Co is larger. Owing to the introduced Co element replacing Fe, the binding energy of the Co—O—V bond is greater than that of Fe—O—V, so V and O simultaneously move in the direction of high binding energy. Figure 2d displays the characteristic peak at 398.43 and 399.93 eV, corresponding to N—C (pyridine nitrogen) and N—Fe of BVO/He, respectively.^[^ ^16^ ^]^ Furthermore, the peak at 710.81 eV corresponds to Fe^2+^ in BVO/He (Figure 2e), in line with the characteristics of He as a ferroporphyrin compound. While after the Co is introduced into the He film, the peaks of N and Fe shift toward higher binding energy. The C 1s of BVO/He and BVO/HC located at 284.56, 285.17, 286.24, and 288.21 eV correspond to C—C, C=C, C=O, and C—N functional groups (Figure S6, Supporting Information), respectively. The shift of these XPS characteristic peaks indicates that Co is introduced by replacing Fe in He and forming a Co—O—V bond with BVO. The formed Co—O—V bond improves the combination of BVO/cocatalyst and facilitates the interfacial transport of photogenerated carriers, which we will discuss as follows.

**Figure 3:**
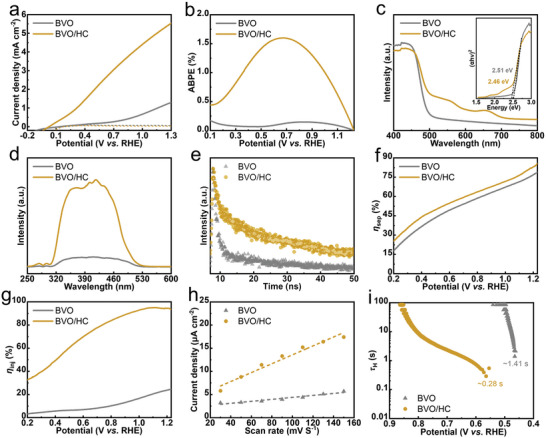
a) Linear sweep voltammogram curves. b) *η*_ABPE_ curves. c) UV–visible absorption spectra (the inset is the corresponding Tauc plots). d) Surface photovoltage spectroscopy. e) Time‐resolved transient photoluminescence decay spectra. f) Charge separation efficiency. g) Charge injection efficiency. h) Double‐layer capacitance. i) Carrier lifetime derived from the OCP decay curve at the transient when illumination is removed at the OC condition and plotted as a function of OCP on a logarithmic scale for BVO and BVO/HC.

The photocurrent‐voltage (*J–V*) curves of BVO/HC with different amounts of Co were presented in Figure S7, Supporting Information. With the Co content increasing, the *J* increases first and then decreases. The optimal performance is achieved for the 0.5 g sample. Compared to BVO, the performance of the BVO/HC photoanode is considerably improved (**Figure** **3**a). The *J* reaches 5.3 mA cm^−2^ at 1.23 V versus RHE, and the *V*_on_ shifts negatively to 0.07 V from 0.45 V in 0.5 m Na_2_SO_4_ aqueous solution. The considerably improved *J* and more negative *V*_on_ indicate that the conformal‐covered HC film acts as an efficient cocatalyst to considerably enhance the performance of BVO. The applied bias photon‐to‐current efficiency (*η*_ABPE_) under different bias voltages is calculated in Figure 3b. The BVO/HC photoanode exhibits a higher *η*_ABPE_ of 1.60% at 0.68 V versus RHE, suggesting the evident performance improvement of BVO/HC over the measured potential range. Figure 3c exhibits the UV–visible absorption curves and corresponding Tauc plots. The BVO/HC exhibits a narrower bandgap (2.46 eV) and stronger absorption in the range of 500–700 nm. Figure S8a,b, Supporting Information shows the optical properties based on transmittance and reflectance spectra; BVO/HC exhibits a slightly improved light harvesting capability. To investigate the effect of enhanced light absorption capacity on performance improvement, the unity converted photocurrent density (*J*_abs_) is calculated from UV–visible absorption spectra and summarized in Table S2, Supporting Information.^[^ ^32^ ^]^ The *J*_abs_ increases from 5.96 to 6.61 mA cm^−2^ after coating with HC films. The slightly increased *J*_abs_ indicates that the light absorption capacity does not impact PEC performance. Additionally, the photovoltage is related to the PEC performance, and the photovoltage can be determined using the open‐circuit potential (OCP) measurement. The OCP in Figure S9a, Supporting Information is measured from the voltage‐time (*V–T*) curves in Figure S9b, Supporting Information. BVO/HC displays a larger OCP value, indicating excellent carrier separation capability. To further explore the effect of HC film on the separation and transport behavior of photogenerated electron‐hole pairs, surface photovoltage spectroscopy (SPV) was performed. BVO/HC exhibits a higher SPV response intensity relative to pure BVO in the whole wavelength range of light absorption (Figure 3d). Time‐resolved photoluminescence decay (TRPL, Figure 3e) spectrum was measured to compare the photogenerated carrier lifetime. Compared with BVO (*τ* = 16.87 ns, Table S3, Supporting Information), BVO/HC (*τ* = 22.27 ns) demonstrates a longer carrier lifetime, slower recombination rate, enhanced carrier transfer efficiency, and decreased recombination. The charge separation efficiency (*η*_sep._) was estimated further to explore the transfer of photogenerated carriers in the photoanode. The detailed calculation process is presented in the supporting information. Figure S10, Supporting Information shows the *J–V* curves measured in the electrolyte of 0.5 m Na_2_SO_3_ containing NaH_2_PO_4_, used as the hole scavenger. The *η*_sep._ of BVO/HC photoanode is higher than that of BVO at 0.2–1.2 V versus RHE (Figure 3f), suggesting enhanced carrier separation. The above results prove that the covering of HC film is beneficial to the separation of photogenerated carriers in bulk and effectively inhibits bulk recombination, promoting the PEC performance of the photoanode to a certain extent.

In addition to the bulk transfer behavior of charge carriers, surface catalysis of photogenerated holes considerably affects the PEC performance. Charge injection efficiency (*η*_inj._) represents the fraction of the holes in water oxidation at the photoanode/electrolyte interface. As shown in Figure 3g, the BVO/HC photoanode exhibits remarkably improved *η*_inj._ compared with BVO over the entire potential range, confirming that the HC film acts as a cocatalyst to increase surface‐active sites and boost the surface OER dynamics. Compared with *η*_sep._, this improved *η*_inj._ indicates that the enhanced performance of BVO/HC is mainly due to the modification of the surface catalytic process. The electrochemically active surface area (ECSA) plots are calculated from the double‐layer capacitance (*C*_dl_) measurements to confirm the increased active sites induced by the HC film.^[^ ^33^ ^]^ Cyclic voltammetry curves were measured in the non‐Faradaic region (0.5–0.6 V vs RHE) with different scan rates (0.03–0.15 V s^−1^) (Figure S11, Supporting Information) to obtain *C*_dl_. Figure 3h reveals that *C*_dl_ for BVO/HC is larger than that for BVO. A more positive slope implies more active reaction sites, which indicates increased surface‐active sites and a larger reaction area of BVO/HC. Their electrochemical OER properties under dark conditions have also been studied and shown in Figure S12, Supporting Information. BVO/HC possesses a lower *η* and higher water oxidation current compared with pristine BVO under dark reaction circumstances, further revealing the excellent OER electrocatalytic activity of BVO/HC and proved the HC films to be an excellent cocatalyst. Furthermore, the hole lifetime (*τ*_H_) can be expected from OCP decay. BVO/HC exhibited a carrier lifetime of 0.28 s at the transient when the illumination was switched off (Figure 3i), which is decreased compared with BVO (*τ*_H_ = 1.41 s). This suggests that the formed cocatalysts promote the transfer of photogenerated holes. The faster decay kinetics upon switching off the illumination suggests the reduced charge trapping at the interfaces in BVO/HC photoanode. These data reveal that the presence of HC film is an excellent cocatalyst to accelerate the transfer of photogenerated holes near the photoanode/electrolyte interface, leading to improved PEC activity.

**Figure 4:**
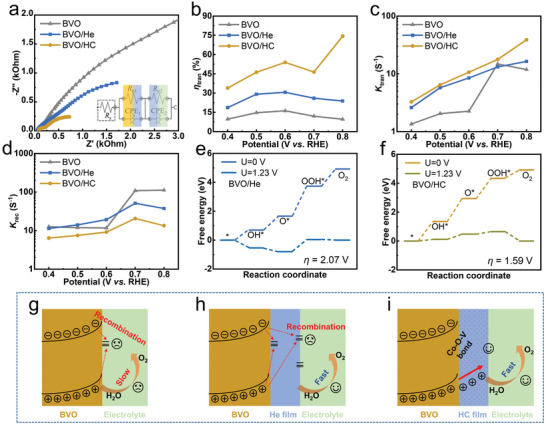
a) Nyquist plots. b) Surface charge‐transfer efficiency. c) Surface charge‐transfer rate constants. d) Surface charge recombination rate constants of BVO, BVO/He, and BVO/HC, respectively. Free energies of OER reaction steps for e) BVO/He and f) BVO/HC systems. The entire structure of the OER four‐step reaction is shown in Figure S15, Supporting Information.

To further explore the role of Co, some related measurements were applied for BVO/He. BVO/He exhibits poor performance (lower *J* and more positive *V*_on_) compared with BVO/HC (Figure S13, Supporting Information), indicating the importance of Co for PEC performance. The electrochemical impedance spectroscopy (EIS) was further characterized, as shown in **Figure** **4**a, to probe the interface reaction dynamics of the photoanode. The inset shows the fitting circuit used to extract the resistances. Unlike BVO, BVO/He, and BVO/HC exhibit two circles, meaning that a new interface (BVO/cocatalysts) is formed. The series resistance (*R*_s_) represents the impedance between fluorine‐doped tin oxide and the photoanode, and the charge‐transfer resistances *R*_p1_ and *R*_p2_ represent the impedance at the interface between BVO/cocatalysts film and the cocatalysts/electrolyte, respectively.^[^ ^34^ ^]^ The specific values are shown in Table S4, c. Compared with BVO/He, BVO/HC shows a smaller *R*_p1_ value (Figure 4a), meaning that the charge carriers can transfer faster at the interface of BVO/cocatalysts. As described before, this is due to the introduced Co replacing Fe in the HC film; the formed Co—O—V bonds exhibited a strong combination between the interface of BVO and HC film, which is beneficial to the interfacial transport of photogenerated carriers and reduces the interface recombination. Additionally, The *R*_p2_ is in the order of BVO > BVO/He > BVO/HC, indicating that the HC film acts as a cocatalyst, reduces the surface impedance, and further facilitates the surface OER kinetics of the photoanode. The smallest value of *R*_p2_ suggested that the introduction of Co further promotes the surface catalytic reaction kinetics. Intensity‐modulated photocurrent spectroscopy (IMPS) kinetic analysis was performed to further investigate the charge‐transfer dynamics processes, including surface charge‐transfer efficiency (*η*_tran_), surface charge‐transfer rate constants (*K*_tran_), and surface charge recombination rate constants (*K*_rec_).^[^ ^35^ ^]^ Therefore, we performed IMPS tests to explore the specific role of HC film in the surface catalytic reaction. The IMPS curves of BVO, BVO/He, and BVO/HC were measured with different voltages (0.4–0.8 V) under *λ* = 365 nm irradiation (Figure S14, Supporting Information). The calculation details of the rate constants are presented in the supporting information. The *η*_tran_ of BVO is unchanged, while BVO/HC has a huge improvement (Figure 4a), according to the trend of *η*_inj._. This confirms that IMPS can be used to analyze surface OER kinetics. Figure 4a reveals that the *η*_tran_ is in the order of BVO/HC > BVO/He > BVO, indicating that the coated He film promotes the transfer of carriers and the introduced Co further accelerates the extraction of holes, consistent with EIS data. For BVO, the values of the *K*_tran_ over the entire voltage range are much smaller than those of the *K*_rec_ (Figure 4c,d), indicating that most of the carriers on the photoanode surface will occur as recombination and not transfer into the electrolyte, resulting in poor performance of BVO. Compared with BVO, the *K*_tran_ of BVO/He is improved while the *K*_rec_ is unchanged, indicating that the presence of the He film not only effectively promotes the transfer of photogenerated holes but also introduces some defects, resulting in the recombination of photogenerated carriers not being effectively inhibited. Once Co is introduced into the He film, BVO/HC displays the largest *K*_tran_ and the smallest *K*_rec_ among the three samples, proving that the Co‐modified film could effectively reduce the recombination and promote the OER reaction kinetics on the photoanode surface. Furthermore, the *K*_tran_ stays unchanged between BVO/He and BVO/HC, while the *K*_rec_ reduces after Co is introduced over the measured potential. This phenomenon indicates that the existence of Co greatly reduces the recombination of photogenerated carriers, which has no obvious effect on the transport rate of carriers. Furthermore, to rationalize the improved catalytic performance of the BVO/HC photoanode and explore the effect of the presence of Co on OER kinetics, we obtained the *η* for OER of the BVO/He and BVO/HC systems based on the DFT calculations to estimate the energy barrier of each OER pathway on BVO/He and BVO/HC photoanodes. The computational details are described in Supporting Information. The Gibbs free energy diagrams calculated on the surface of BVO/He and BVO/HC photoanodes are shown in Figure 4e,f. The detailed Gibbs free energy of each step is presented in Table S5, Supporting Information. The *η* decreases to 0.36 V in the BVO/HC from 0.84 V in the BVO/He (Figure 4e,f). The rate‐determining step (RDS) of BVO/He is step 3 (O* to OOH*) with a high *η* of 0.84 V, while the first two steps require relatively low energies. The notable 0.48 V drop in *η* provides solid evidence that the catalytic performance is enhanced in BVO/HC photoanode once the Co is introduced because the Co replaces Fe in this film and alters the RDS of *O to *OOH (step 3) in the BVO/He system (Figure 4e) into *OH to *O (step 2) in the BVO/HC (Figure 4f) system, leading to that the OOH* is preferred for formation on the BVO/HC catalyst and favors O_2_ production. The DFT calculations provide an excellent explanation for the experimental results. These data effectively indicate that although the presence of the He film promotes the transfer of photogenerated holes, some defects are simultaneously introduced, leading to severe carrier recombination (Figure 4g,h). Further, the introduced Co—O—V bond is favorable for the interfacial transport of photogenerated carriers and reduces the recombination of the carriers. Simultaneously, the modification of Co into He film greatly reduces the surface defects and recombination of carriers, revealing that the formed HC film is favorable for hole extraction and lowers the surface OER reaction barrier of the photoanode (Figure 4i). Therefore, the HC films are critical for enhanced OER reaction.

## 3 Conclusion

In summary, a simple solvothermal‐assisted process is utilized to introduce large conjugated ring structure‐rich cocatalyst films onto the surface of the BVO photoanode, which acts as an efficient cocatalyst. The BVO/HC photoanode exhibits excellent PEC performance, with the *V*_on_ shifting negatively to 0.07 V and the *J* increasing to 5.3 mA cm^−2^ at 1.23 V versus RHE. The enhancement mechanism is discussed and mainly attributed to enhanced surface OER dynamics. The unexpectedly formed Co—O—V bond at the photoanode/cocatalyst interface enhances the combination of photoanode/cocatalyst. It reduces carrier recombination, which is beneficial to transporting photogenerated holes. Furthermore, the experimental and theoretical calculations certify that the covered HC film acts as an efficient cocatalyst, effectively facilitates the transfer of holes, reduces the surface *η*, and boosts the kinetics of OER by precisely affecting the forming processes of OOH*. Our work provides a facile and efficient strategy to simultaneously address sluggish OER kinetics and severe carrier recombination in photoanodes.

## 4 Experimental Section

### Synthesis of BiVO_4_ Photoanode

BVO photoanode was prepared using modified electrodeposition procedures on fluorine‐doped tin oxide (FTO) glass.^[^ ^18^ ^]^ FTO was first sonicated three times with acetone, ethanol, and deionized water for 10 min each. Then, the precursor solution was prepared through the following steps. A solution: 23.904 g potassium iodide (KI) was dispersed in 360 mL deionized water; then, 7.056 g bismuth nitrate pentahydrate [Bi(NO_3_)_3_∙5H_2_O] was added, and the pH was adjusted to 1.7 by adding nitric acid (HNO_3_). B solution: 3.577 g benzoquinone was dissolved in 144 mL ethyl alcohol absolute (C_2_H_5_O). The two solutions were followed by stirring for 2 h. The steps of electrochemical deposition are as follows: FTO was used as the working electrode, Ag/AgCl was used as the reference electrode, and Pt mesh as a counter electrode. The cathodic deposition was performed under −0.1 V versus Ag/AgCl with 180 s and obtained iodine bismuth oxide (BiOI). Finally, 0.53 g vanadyl acetylacetonate [VO(acac)_2_] with 10 mL dimethyl sulfoxide (DMSO) solution was prepared. Then, the above 300 µL solution was placed on the as‐fabricated BiOI and heated at 450 °C for 2 h in a muffle furnace. The excess vanadium pentoxide (V_2_O_5_) present in the BVO film was removed by soaking in 1 mol L^−1^ NaOH solution for 30 min; the BVO was formed and rinsed with absolute ethanol, then stored in a vacuum oven.

### Synthesis of BVO/He Photoanode

The BVO/He photoanode was prepared through a solvothermal process. Then, the precursor solution was prepared through the following steps: 0.2 g heme was dissolved into 200 mL *N*,*N*‐dimethylformamide (DMF), followed by stirring for several minutes. The as‐prepared BVO was then placed in a 25 mL Teflon‐lined stainless steel autoclave. Each lining was pipetted with 10 mL of the prepared precursor solution; the vessel was then sealed and heated up to 70 °C for 12 h. After cooling to room temperature, the BVO/He was formed and rinsed with absolute ethanol, then stored in a vacuum oven.

### Synthesis of BVO/HC Photoanode

The same solvothermal procedure was performed to synthesize BVO/HC photoanode except that the additional 0.5 g Cobaltous nitrate [Co(NO_3_)_2_] was added to the abovementioned precursor solution.

## Conflict of Interest

The authors declare no conflict of interest.

## Supporting information

Supporting Information

## Data Availability

The data that support the findings of this study are available from the corresponding author upon reasonable request.
