# Supplementary material for: Porphyrins‐Assisted Cocatalyst Engineering with Co—O—V Bond in BiVO4 Photoanode for Efficient Oxygen Evolution Reaction
Source: Adv Sci (Weinh). 2023 Jan 16;10(8):2206729. doi: 10.1002/advs.202206729 (PMC10015896; doi:10.1002/advs.202206729)
Supplement: Supplementary file 1 — Supporting Information [file ADVS-10-2206729-s001.pdf]

WILEY-VCH

Supporting Information

Porphyrins-Assisted Cocatalyst Engineering with Co–O–V Bond in $BiVO_4$ Photoanode for Efficient Oxygen Evolution Reaction

*Linxing Meng, Zunyan Lv, Weiwei Xu, Wei Tian, and Liang Li**

Experimental Section

Materials Characterization. Scanning electron microscopy (SEM, Regulus 8100, Hitachi Limited, Japan) and Transmission electron microscopy (TEM, Tecnai G2 F20 200 KV, FEI, USA) were employed to determine the morphology of samples. The element distribution was conducted using energy-dispersive X-ray spectroscopy (EDX) under the TEM with an annular dark-field detector. X-ray diffraction (XRD) patterns (D8 ADVANCE, Bruker, Germany) and Raman spectroscopy (Xplora Plus, Horiba, Japan) were managed to characterize the phase of the samples. The X-ray photoelectron spectroscopy (XPS) measurement (ESCALAB 250Xi, Thermo Fisher Scientific, USA) was performed to verify the element chemical status. The light absorption of the samples was recorded to characterize the light absorption of the sample using a UV-vis spectrophotometer (UV-3600, SHIMADU, Japan). A LifeSpec II with a 450 nm laser (FLS1000, Edinburgh instruments, UK) was employed to measure the time-resolved transient photoluminescence decay (TRPL) spectra (wavelength width: ±10 nm, maximum average power: 5 mV). The fitted carrier lifetime data were calculated according to $\tau = \sum A_n\tau_n^2/\sum A_n\tau_n$ (n = 1, 2, 3…). The surface photovoltage spectroscopy (SPV, PerfectLifht, China) was performed to explore the separation and transport behavior of photogenerated electron-hole pairs.

Photoelectrochemical and Electrochemical Measurements. The electrochemical workstation (PGSTAT 302N, Autolab, Switzerland) with a three-electrode system (Ag/AgCl reference electrode and a Pt mesh counter electrode) was used for all measurements, and the electrolyte was 0.5 M Na_2SO_4 with pH ≈ 6.8. The photocurrent-voltage (*J-V*) curves were measured from the positive to the negative direction at a scan rate of 0.01 V s^{-1} under AM 1.5 G illumination (100 mW cm^{-2}) from a solar light simulator (94043A, Newport, USA). Electrochemical impedance spectra (EIS) were

performed with frequencies ranging from 0.1 Hz to 100 kHz at an open-circuit voltage under light illumination.

With the Nernst equation, the measured potential versus Ag/AgCl was converted to the reversible hydrogen electrode (RHE):[1]

$$E_{RHE} = E_{Ag/AgCl} + 0.059\ pH + E^{o}_{Ag/AgCl} \tag{1}$$

where $E^{o}_{Ag/AgCl}$ = 0.1976 V at 25 °C and $E_{Ag/AgCl}$ is the experimentally measured potential versus Ag/AgCl reference.

An electrochemical surface area (ECSA) was determined by measuring the double-layer capacitance (C_{dl}), which was obtained by deriving the cyclic voltammetry curves with different scan rates.[2] At different scan rates (0.03-0.15 V s^{-1}), the potential was swept in a non-Faradaic region (0.5-0.6 V *vs.* RHE). The measured capacitive currents were plotted as a scan rate function.

The applied bias photon-to-current efficiency (η_{ABPE}) was calculated from the *J*-*V* curves using the following equation:[3]

$$\eta = \frac{(J_{\mathrm{light}} - J_{\mathrm{dark}})(mA \times cm^{-2}) \times (1.23 - V_{\mathrm{RHE}})\ (V)}{P_{\mathrm{sunlight}}\ (mW \times cm^{-2})} \times 100\% \tag{2}$$

where V_{RHE} is the potential of the working electrode versus the RHE, J_{dark} and J_{light} are the measured current density in the dark and under illumination, respectively, and $P_{sunlight}$ is the incident light power (100 mW cm^{-2}).

The unity converted photocurrent density ($J_{abs.}$) is calculated using $\eta_{abs.}$ over the standard solar spectrum:[4]

$$J_{\mathrm{abs.}} = \int_{300}^{\lambda\max} \frac{\lambda \times J_{abs.}(\lambda) \times E(\lambda)}{1240} d(\lambda) \tag{3}$$

where λ_{max} is the maximum light absorption edge of a photoelectrode, λ (nm) is the light wavelength, and *E*(λ) is the power density (mW cm^{-2}) at a specific wavelength (λ) of the standard solar spectrum.

The charge separation efficiency ($\eta_{sep.}$) and injection efficiency ($\eta_{inj.}$) were measured in the 0.5 M Na_2SO_3 containing NaH_2PO_4 electrolyte as a hole scavenger. $\eta_{sep.}$ is defined as the fraction of the photogenerated holes reaching the electrode/electrolyte interface, reflecting the recombination rate and separation capability of carriers in bulk. $\eta_{inj.}$ is the fraction of those holes at the photoanode/electrolyte interface used for water oxidation. Thus, $\eta_{inj.}$ would be enhanced by reducing surface recombination and by increasing the OER of holes. The $\eta_{sep.}$ and $\eta_{inj.}$ were calculated using the equations:[5]

$$\eta_{inj.} = J/J_{sulfite} \quad (4)$$

$$\eta_{sep.} = J_{sulfite}/J_{abs} \quad (5)$$

where $J_{sulfite}$ represents the photocurrent density tested in the hole scavenger.

The photogenerated carrier lifetime calculation obtained from the open-circuit potential (OCP) measurements is as follows:[6]

$$\tau = -\frac{k_n T}{e}\left(\frac{d_{OCP}}{dt}\right)^{-1} \quad (6)$$

where τ is the carrier lifetime, k_B is Boltzmann's constant, T is the temperature, and e is the positive charge.

The intensity-modulated photocurrent spectroscopy (IMPS) kinetic analysis was performed on an electrochemical workstation (CIMPS, Zennium Zahner) similar to PEC measurements. The irradiation source is an LED (s/n Ls 1272, Zennium Zahner) with 365-nm wavelength, and the power intensity is 450 W m^{-2}, and the ±5% modulation intensity is ~450 W m^{-2}. The Nyquist plots were used to get the surface charge-transfer and recombination rate constants.

Computational Details. DFT calculations were employed to estimate the energy barrier of each OER pathway using the Dmol^3 code on BVO/He and BVO/HC photoanodes.[7] The exchange-correlation interaction was treated by the generalized gradient approximation with PBE functional.[8] A double numerical quality basis set with a d-

type polarization function (DNP) was utilized for all the geometric optimizations,[9] and total energy calculations. Grimme's semiempirical DFT-D was introduced in the computations to guarantee a better description of the electron interaction in a long range.[10] The core electrons were modeled using effective core pseudopotentials by Dolg[11] and Bergner.[12] All calculations were spin unrestricted. The positions of all atoms were fully relaxed until the following convergence criterion was met: 0.002 Ha/Å for force, 10^{-5} Ha for total energy, and 0.005 Å for displacement. The actual space cutoff radius was 4.1 Å. The self-consistent field computations criterion was chosen to be 10^{-6} Ha. The Gibbs free energy diagrams calculated on the surface of BVO/He and BVO/HC photoanodes are shown in Figures 4e,f. The detailed Gibbs free energy of each step is presented in Table S5 (Supporting Information) according to the above formula above.

The OER processes on the BVO/He and BVO/HC were described as the adsorption of successive intermediate species on the catalyst, and the relevant reaction energies were as described using equations S1-S4

$$OH^{-} + cat \rightarrow *OH\text{-}cat + e^{-} \quad (S1)$$

$$*OH\text{-}cat + OH^{-} \rightarrow *O\text{-}cat + H_2O + e^{-} \quad (S2)$$

$$*O\text{-}cat + OH^{-} \rightarrow *OOH\text{-}cat + e^{-} \quad (S3)$$

$$*OOH\text{-}cat + OH^{-} \rightarrow O_2\uparrow + H_2O + e^{-} \quad (S4)$$

In the above reactions, the "cat" represented the active site when OER occurred. The *O, *, and *OO represented the intermediate species adsorbed on the active sites.

To evaluate OER activity, we calculated the free energy (ΔG_1-ΔG_4) using the computational standard hydrogen electrode model. The free energy calculation could be obtained using Equations G1-G4:

$$\Delta G_1 = G_{OH\text{-}cat} - G_{cat} - G_{H2O} + 1/2G_{H2} - eU + K_BTLn10 \cdot pH \quad (G1)$$

$\Delta G_2 = G_{O\text{-}cat} - G_{OH\text{-}cat} + 1/2G_{H2} - eU + K_BTLn10{\cdot}pH$ (G2)

$\Delta G_3 = G_{OOH\text{-}cat} - G_{O\text{-}cat} - G_{H2O} + 1/2G_{H2} - eU + K_BTLn10{\cdot}\ pH$ (G3)

$\Delta G_4 = 4.92 - \Delta G1 - \Delta G2 - \Delta G3$ (G4)

It should be noted that $-eU$ represented the free energy changes for one electron transfer where U was electrode potential concerning the standard hydrogen electrode. For pH $\neq$ 0, pH affected by free energy could be defined as $-K_BTLn10{\cdot}pH$, where K_B was the Boltzman constant. ΔG_4 was calculated using $4.92 - \Delta G1 - \Delta G2 - \Delta G3$ to avoid calculating the O_2 adsorption and desorption. It was known that the DFT calculation might not accurately describe the free energy of the O_2 molecule in the gas phase; hence, we used H_2O and H_2 as a reference and extracted the free energy of O_2 through the reaction $O_2 + 4(H^+ + e^-) \rightarrow 2H_2O$. The equilibrium potential for this reaction was 1.23 V; since it was a four-electron transfer reaction, the full energy was 4 × 1.23 = 4.92 eV. Then, the overpotential (η) of OER can be computed based on the following formula:

$$\eta = \max\left(\frac{\Delta G_{\mathrm{OER}}}{e}\right) - 1.23\ \mathrm{V} \quad (7)$$

Supporting Figure Captions

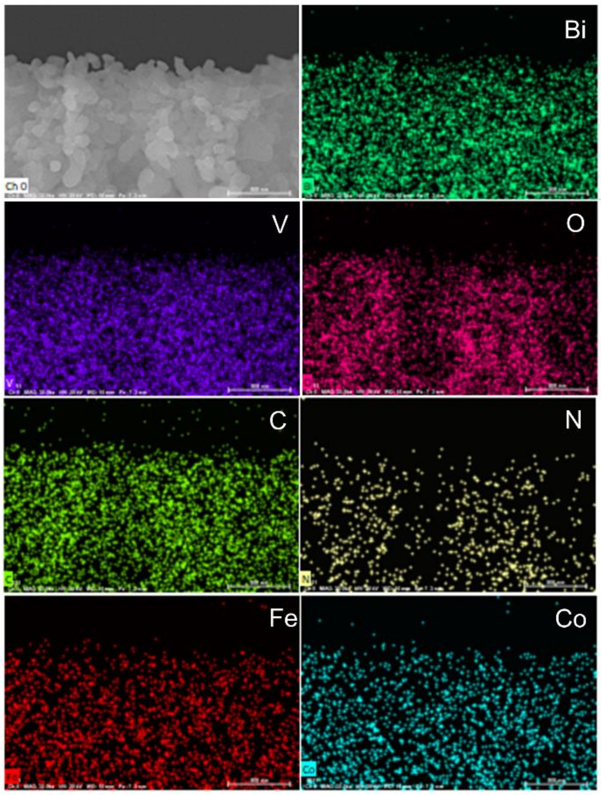

Figure S1. Elemental mapping of BVO/HC under SEM.

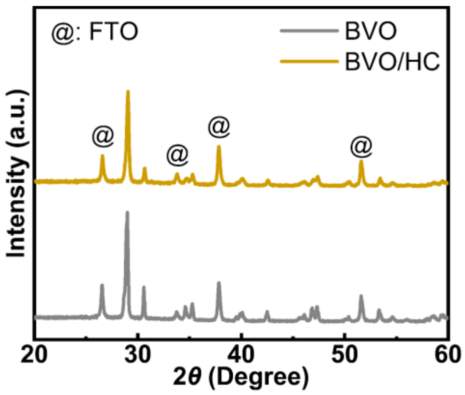

Figure S2. XRD pattern of BVO and BVO/HC.

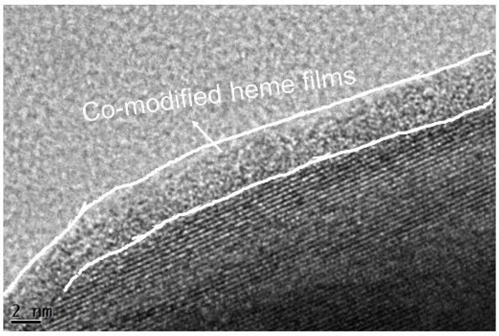

Figure S3. HRTEM image of BVO/HC.

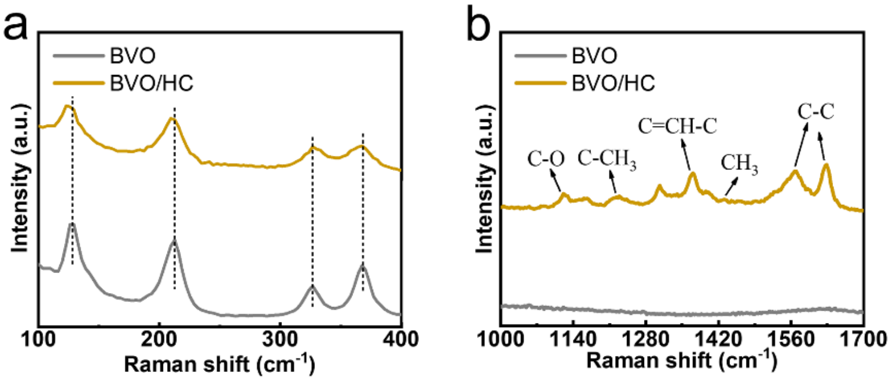

Figure S4. Raman spectra of a) BVO and b) BVO/HC.

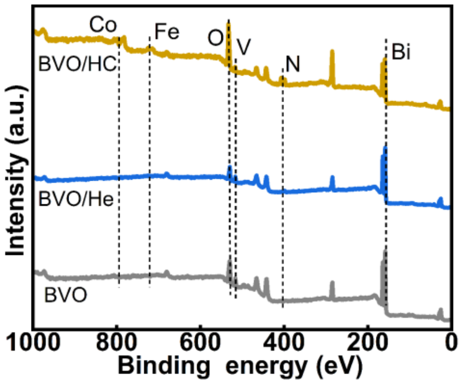

Figure S5. XPS survey spectra of BVO, BVO/He, and BVO/HC.

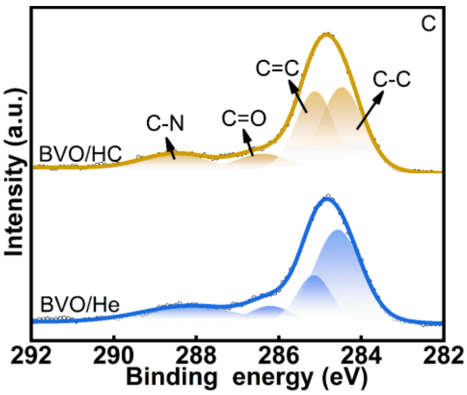

Figure S6. XPS spectra for O 1s of BVO/He and BVO/HC.

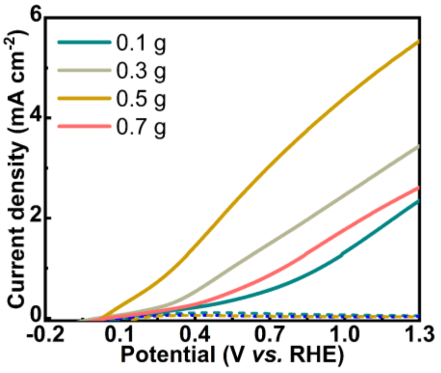

Figure S7. Linear sweep voltammogram curves of BVO/HC photoanodes with different contents of Co measured in 0.5 M Na_2SO_4.

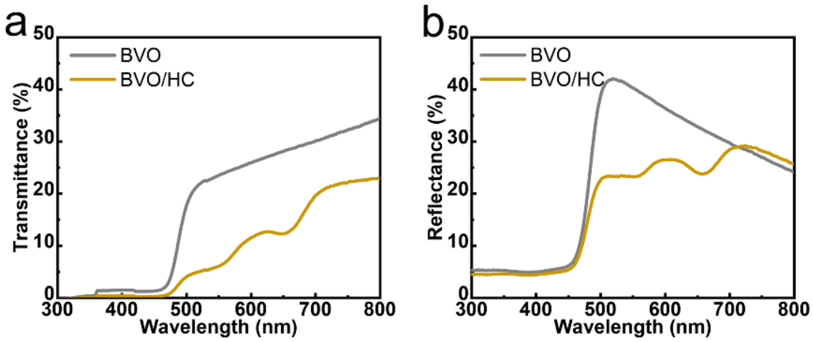

Figure S8. a) Transmittance spectra and b) reflectance spectra of BVO and BVO/HC.

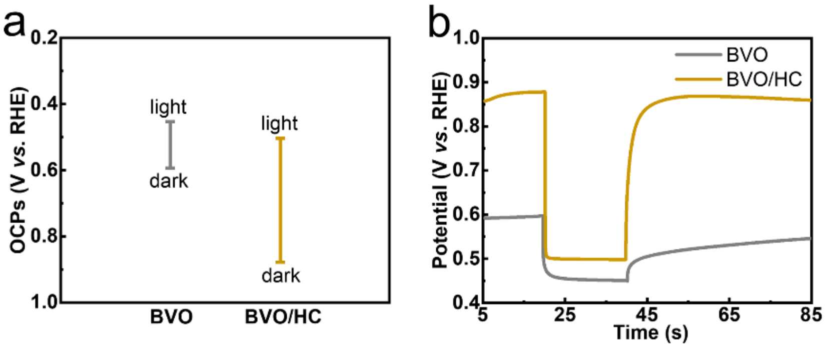

Figure S9. a) OCP values of BVO and BVO/HC photoanodes and b) OCP measurements under dark and light conditions.

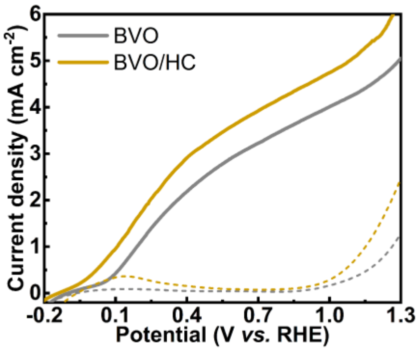

Figure S10. Linear sweep voltammogram curves of BVO and BVO/HC photoanodes. 0.5 M Na_2SO_3 containing NaH_2PO_4 is used as a hole scavenger.

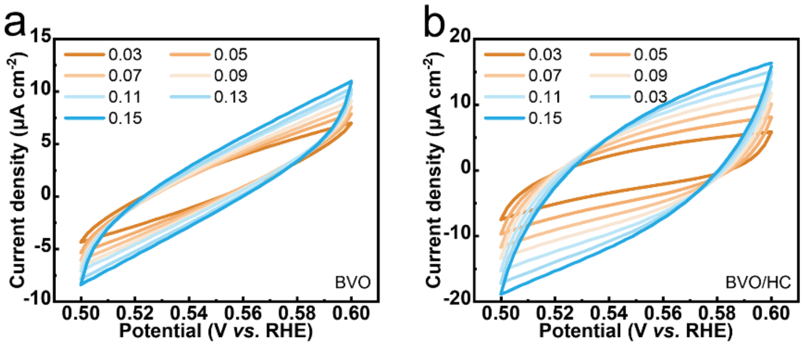

Figure S11. Cyclic voltammograms of a) BVO. b) BVO/HC photoanodes were measured in a non-Faradaic region (0.5-0.6 V *vs.* RHE) at the scan rates: 0.03, 0.05, 0.07, 0.09, 0.11, 0.13, and 0.15 V s^{-1}.

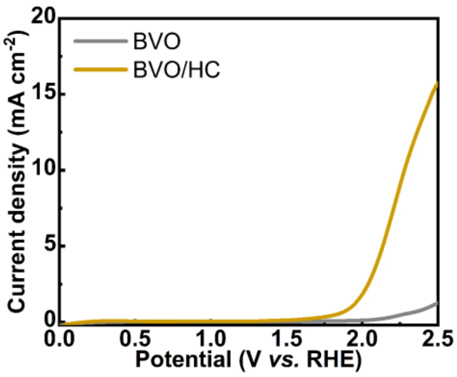

Figure S12. Linear sweep voltammogram curves of BVO and BVO/HC photoanodes under dark measured in 0.5 M Na_2SO_4.

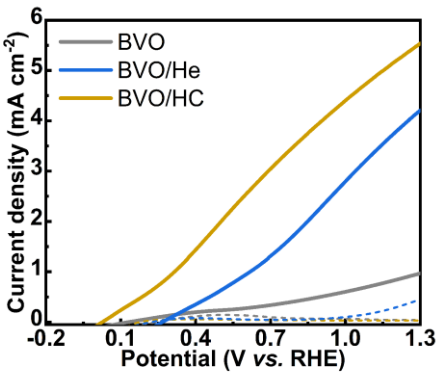

Figure S13. Linear sweep voltammogram curves of BVO, BVO/He, and BVO/HC measured in 0.5 M Na_2SO_4.

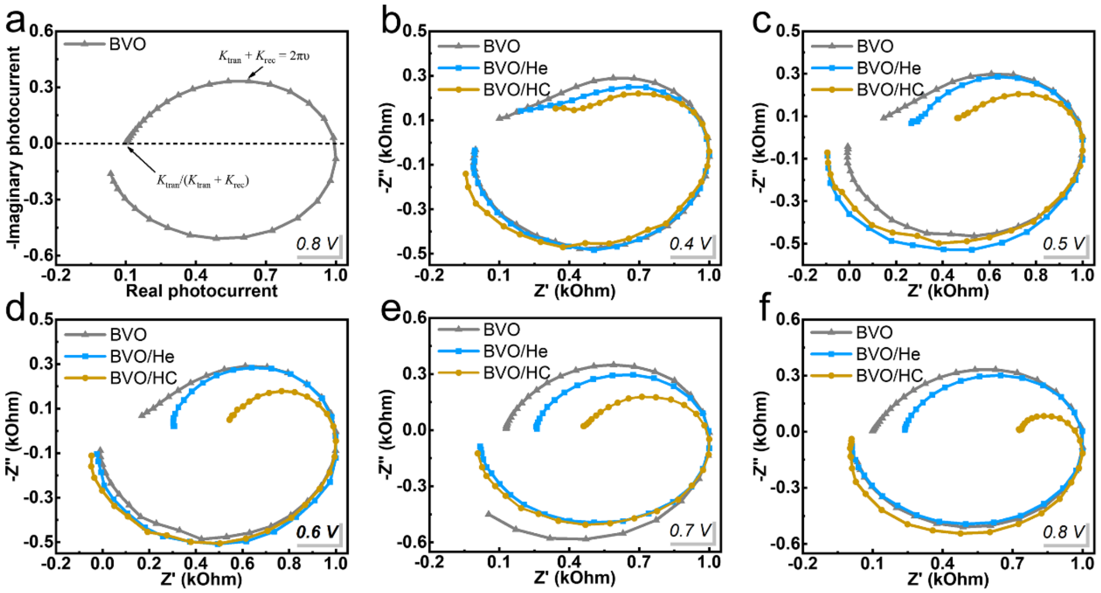

Figure S14. a) IMPS results and the detailed calculation equation. IMPS plots of BVO, BVO/He, and BVO/HC at a different potential: b) 0.4, c) 0.5, d) 0.6, e) 0.7, and f) 0.8 V.

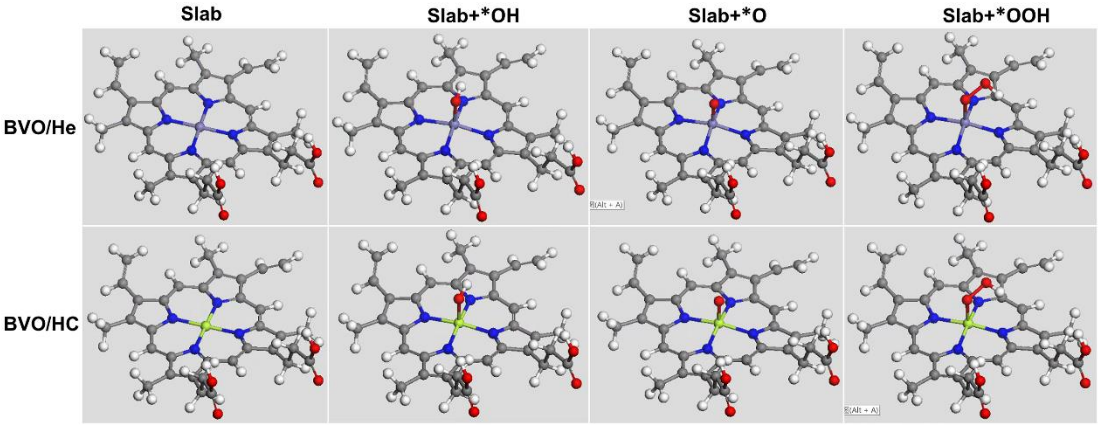

Figure S15. All optimized structures of the oxygen evolution four-step reaction for the BVO/He and BVO/HC systems.

Table S1. J of BVO/HC in comparison with other BVO-based photoanodes.

Photoelectrode materials	Electrolyte	V_{on} (V *vs.* RHE)	*J* @ 1.23 V *vs.* RHE (mA cm^{-2})	References
BVO/Co-Si	Na_2SO_4	0.18	5.00	[13]
BVO/Co_3O_4/FeOOH/ NiOOH	Na_2SO_4	0.16	4.50	[14]
BVO/La:$BaSnO_3$	Na_2SO_4	0.32	5.15	[15]
BVO/CoSn	Na_2SO_4	0.35	4.15	[16]
BVO/Co@CB	Na_2SO_4	0.15	4.80	[17]
BVO/P3HT	Na_2SO_4	0.18	4.25	[18]
BVO/EL	Na_2SO_4	0.23	4.60	[19]
BVO/V_{Bi}	Na_2SO_4	0.30	4.50	[20]
BVO/B	Na_2SO_4	0.22	3.50	[21]
BVO/BP/OEC	Na_2SO_4	0.15	4.48	[22]
BVO/N-CFO	Na_2SO_4	-	4.83	[23]
BVO/HC	Na_2SO_4	0.07	5.30	This work

Table S2. The unity converted photocurrent density (J_{abs}) of BVO and BVO/HC, which is calculated from UV-visible absorption spectra.

	J_{abs} (mA cm^{-2})
BVO	5.96
BVO/HC	6.61

Table S3. The summary of fitted lifetime from the TRPL decay spectra.

	A_1/%	τ_1/ns	A_2/%	τ_2/ns	τ/ns
BVO	22.48	1.09	77.52	21.44	16.87
BVO/HC	5.52	1.58	94.48	23.48	22.27

Table S4. EIS fitted parameters of samples shown in this work.

	R_s (Ω)	R_{p1} (Ω)	R_{p2} (Ω)
BVO	84.6	-	8.5k
BVO/He	74.2	395	2.4k
BVO/HC	80.8	104	1.1k

Note: The equivalent circuit parameters include the series resistance (R_s) between FTO and samples and charge-transfer resistance (R_{p1} and R_{p2}), which means the impedance at the interface between BVO/cocatalyst and the cocatalyst/electrolyte, respectively.

Table S5. Free energy changes four elementary steps for OER in BVO/He and BVO/HC systems when the applied potential is 0 or 1.23 V, and the free energy unit is eV.

	U (V)	Step 1	Step 2	Step 3	Step 4
BVO/He	0	0.69	0.97	2.07	1.19
	1.23	−0.54	−0.26	0.84	−0.04
BVO/HC	0	1.35	1.59	1.40	0.58
	1.23	0.12	0.36	0.17	−0.65

Supporting reference

[1] J. J. Gao, H. B. Tao, B. Liu, *Adv. Mater.* **2021**, 2003786.

[2] L. X. Meng, S. Y. Wang, F. R. Cao, W. Tian, R. Long, L. Li, *Angew. Chem. Inter. Ed.* **2019**, *58*, 6761.

[3] B. Giri, M. Masroor, T. Yan, K. Kushnir, A. D. Carl, C. Doiron, H. C. Zhang, Y. Y. Zhao, A. McClelland, G. A. Tompsett, D. W. Wang, R. L. Grimm, L. V. Titova, P. M. Rao, *Adv. Energy Mater.* **2019**, *9*, 1901236.

[4] T. T. Yao, R. T. Chen, J. J. Li, J. F. Han, W. Qin, H. Wang, J. Y. Shi, F. T. Fan, C. Li, *J Am. Chem. Soc.* **2016**, *138*, 13664.

[5] J. Fu, Z. Y. Fan, M. Nakabayashi, H. X. Ju, N. Pastukhova, Y. Q. Xiao, C. Feng, N. Shibata, K. Domen, Y. B. Li, *Nat. Commun.* **2022**, *13*, 729.

[6] Z. Y. Wang, H. M. Li, S. S. Yi, M. Z. You, H. J. Jing, X. Z. Yue, Z. T. Zhang, D. L. Chen, *Appl. Catal. B:Environ.* **2021**, *297*, 120406.

[7] B Delley, *J. Chem. Phys.* **2000**, *113*, 7756.

[8] J. P. Perdew, K. Burke, M. Ernzerhof, *Phys. Rev. Lett.* **1997**, *77*, 3865.

[9] B. Delley, *J. Chem. Phys.* **1990,** *92*, 508.

[10] S. Grimme, J. Antony, S. Ehrlich, H. Krieg, *J. Chem. Phys.* **2010**, *132*, 154104.

[11] M. Dolg, U. Wedig, H. Stoll, H. Preuss, *J. Chem. Phys.* **1987**, *86*, 154104.

[12] A. Bergner, M. Dolg, W. Küchle, H. Stoll, H. Preuß, *Mol. Phys.* **1993**, *80*, 1431.

[13] Q. Sun, T. Cheng, Z. R. Liu, L. M. Qi, *Appl. Catal. B Environ.* **2020**, *277*, 119189.

[14] B. He, S. R. Jia, M. Y. Zhao, Y. Wang, T. Chen, S. Q. Zhao, Z. Li, Z. Q. Lin, Y. L. Zhao, X. Q. Liu, *Adv. Mater.* **2021**, *33*, 2004406.

[15] J. Jian, Y. X. Xu, X. K. Yang, W. Liu, M. S. Fu, H. W. Yu, F. Xu, F. Feng, L. C. Jia, D. Friedrich, R. van de Krol, H. Q. Wang, *Nat. Commun.* **2019**, *10*, 2609.

[16] S. Alam, M. Qureshi, *J. Phys. Chem. Lett.* **2021**, *12*, 8947.

[17] F. S. Li, H. Yang, Q. M. Zhuo, D. H. Zhou, X. J. Wu, P. L. Zhang, Z. Y. Yao, L. C. Sun, *Angew. Chem. Int. Ed.* **2021**, *60*, 1976.

[18] H. Pei, S. R. Xu, Y. Y. Zhang, Y. Zhou, R. J. Li, T. Y. Peng, *Appl. Catal. B Environ.* **2022**, *318*, 121865.

[19] R. T. Gao, L. Wang, *Angew. Chem. Int. Ed.* **2020**, *59*, 23094.

[20] Y. Lu, Y. L. Yang, X. Y. Fan, Y. Q. Li, D. H. Zhou, B. Cai, L. Y. Wang, K. Fan, K. Zhang, *Adv. Mater.* **2022**, *34*, 2108178.

[21] Q. J. Meng, B. B. Zhang, L. Z. Fan, H. D. Liu, M. Valvo, K. Edstrom, M. Cuartero, R. de Marco, G. A. Crespo, L. C. Sun, *Angew. Chem. Int. Ed.* **2019**, *58*, 19027.

[22] K. Zhang, B. J. Jin, C. Park, Y. Cho, X. F. Song, X. J. Shi, S. L. Zhang, W. Kim, H. B. Zeng, J. H. Park, *Nat. Commun.* **2019**, *10*, 2001.

[23] J. Y. Lin, X. J. Han, S. Y. Liu, Y. Lv, X. Li, Y. X. Zhao, Y. Li, L. Z. Wang, S. M. Zhu, *Appl. Catal. B Environ.* **2023**, *320*, 121947.
